# Cost and cost-effectiveness of alternative screening and diagnostic pathways for achieving hepatitis C elimination in the country of Georgia

**DOI:** 10.1371/journal.pgph.0007122

**Published:** 2026-09-22

**Authors:** Josephine G. Walker, Irina Tskhomelidze Schumacher, Shaun Shadaker, Tamar Gabunia, Rania A. Tohme, Akaki Abutidze, Vladimer Getia, Peter Vickerman

**Affiliations:** 1 Bristol Medical School, University of Bristol, Bristol, United Kingdom; 2 Task Force for Global Health, Tbilisi, Georgia; 3 Division of Viral Hepatitis, US Centers for Disease Control and Prevention, Atlanta, Georgia, United States of America; 4 Ministry of Internally Displaced Persons from Occupied Territories, Health, Labour and Social Affairs, Tbilisi, Georgia; 5 AIDS & Clinical Immunology Research Center, Tbilisi, Georgia; 6 National Center for Disease Control and Public Health, Tbilisi, Georgia; PLOS: Public Library of Science, UNITED STATES OF AMERICA

## Abstract

We evaluated the cost and cost-effectiveness of alternative screening pathways during 2018–2022 within Georgia’s hepatitis C elimination program, which started in 2015. We calculated patient-level costs (2022 USD$) of hepatitis C treatment with centralized and decentralized diagnostic testing in hospitals, primary healthcare (PHC), harm reduction providers (HRP), or specialized providers (SP) from reimbursement records including the value of donated direct-acting antivirals (DAAs). We model hepatitis C case-finding, transmission, and progression over a 20-year time horizon to project cost-effectiveness of treatment for each screening pathway in terms of cost per quality adjusted life year (QALY), compared to a willingness-to-pay threshold of $1,337. Unit costs of treatment decreased from $3942–$4247 across screening pathways in 2018 to $300–$338 in 2022, primarily due to reductions in DAA costs. The cascade of care varied by screening pathway, with highest hepatitis C virus (HCV) antibody prevalence and percent linked to viremia testing among HRP and SP, while total number of patients screened was highest in hospitals. While DAA costs decreased, the cost of case finding increased during 2018–2022, with the biggest increase in hospital settings mainly due to decreasing yield. The program was not cost-effective with full DAA costs, but excluding DAA costs or using lower 2022 costs make all pathways cost-effective and SP, HRP, and PHC potentially cost-saving. Donated drugs allowed Georgia’s HCV elimination program to be cost-effective, while future programs with generic drug costs are likely to be cost-effective. Reductions in yield from hospital screening suggest that later stages of elimination programs should prioritise targeted pathways.

## Introduction

In 2015, a national survey found the prevalence of hepatitis C virus (HCV) infection in Georgia was among the highest in the world. Among adults, 7.7% had antibodies to HCV and 5.4% had chronic hepatitis C, resulting in an estimated 150,000 (95% confidence interval (CI): 128,060 to 173,060) cases [1]. Alongside the national survey in 2015, Georgia adopted a national hepatitis C elimination program, which aims to achieve the elimination of hepatitis C as a public health threat through implementing a comprehensive screening and treatment program [2]. The original program goals were to diagnose 90% of infections, treat 95% of those diagnosed, and cure 95% of treated patients by 2020 [3,4], In addition, the Global Health Sector Strategy to eliminate hepatitis C as a public health threat aims to reduce chronic hepatitis C incidence by 80% and hepatitis C mortality by 65% by 2030 [5].

As of 1 January 2023, over 2.8 million people had been tested at least once for HCV antibodies (anti-HCV) in Georgia, out of a population of 3.7 million. A total of 80,997 people had initiated treatment, with a cure rate of 96.9% among those tested for sustained virological response (SVR) after their first round of treatment. A repeat national sero-survey conducted in 2021, after over 70,000 persons were treated, demonstrated a large decrease in prevalence of chronic hepatitis C to 1.8% among adults (6.8% anti-HCV prevalence); modelling estimates a 58% decrease in incidence of HCV infection during 2015–2022 based on serosurvey prevalence results [6,7].

A previous study estimated that the early stage of the hepatitis C elimination program, through November 2017, was likely to be cost-effective for the government of Georgia, as direct-acting antivirals (DAAs), which were expensive at that time, were donated for free and continue to be donated to the program [8]. Since 2020, generic DAAs have been available globally at low cost, indicating that the program, including paying for DAAs, could be cost effective even at a low willingness-to-pay (WTP) threshold. In this study, we aim to calculate the cost per patient treated based on screening observed at different sites, accounting for differences in anti-HCV prevalence and loss to follow up along the care cascade. We explore how the cost of case finding and treatment has changed over time. We then calculate the cost per quality-adjusted life year (QALY) gained for each of the screening strategies during 2018–2022, by projecting the long-term impact of each treatment in a dynamic model of HCV transmission and disease progression in Georgia. Finally, we explore how changes in costs and prevalence yield could affect the cost-effectiveness of the screening strategies.

## Methods

We used government expenditure records and programmatic data on the hepatitis C care cascade from the elimination program within a decision tree model to estimate the cost per patient treated for alternative screening strategies used in Georgia during 2018–2022. We account for differences in prevalence yield in testing and levels of linkage to viremia testing and treatment across screening settings. We then use a dynamic model to estimate the incremental cost-effectiveness ratio for each pathway, in terms of cost per QALY gained, compared to if each strategy was not included in the elimination program. As Georgia does not have a national cost-effectiveness threshold, we compare estimates to a conservative opportunity-cost based WTP threshold of 20% GDP per capita ($1,337 USD) in 2022, and secondarily to a GDP per capita threshold ($6,685) [9].

### Screening strategies

Routine screening for anti-HCV takes place in a variety of settings in Georgia, including both hospital and community-based settings. The five screening strategies evaluated in this study are at 1) centralized hospital, 2) decentralized hospital, 3) primary healthcare (PHC), 4) harm reduction providers (HRP), and 5) specialized treatment sites (Table 1). Under all pathways, if active infection is diagnosed (detected HCV RNA or positive HCV core antigen), patients are referred for treatment at their preferred location (PHC, HRP, or specialized treatment site).

**Table 1:** Summary of screening pathways for hepatitis C, including number of people screened, number of people treated after diagnosis at that screening site, source of anti-HCV rapid tests, and diagnostic tests used, during 2018–2022 in Georgia.

Screening pathway	Description	N people screened 2018–2022	N treatments 2018–2022	Anti-HCV (Hepatitis C Rapid Test) test source	Diagnostic test used
Centralized hospital	All hospital inpatients were tested for anti-HCV on site, and samples (remaining blood where possible) from anti-HCV positive patients were submitted to the central national reference laboratory (Lugar Center) for viremia testing	667,124	4,233 (0.6%)	Privately purchased	HCV Core Antigen (HCV coreAg), with negative samples and those in the grey zone retested by PCR for HCV RNA to confirm results
Decentralized hospital	Inpatients at participating hospitals were tested for anti-HCV on site, and samples from anti-HCV positive patients were submitted to selected specialized HCV provider labs in Tbilisi, where HCV RNA testing was conducted	700,212	5,632 (0.8%)	Privately purchased	HCV RNA by point of care test (GeneXpert*) or laboratory-based PCR
Primary healthcare	Rapid tests were used to test for anti-HCV, and positive samples were sent to contractor laboratories for viremia testing using either GeneXpert or standard qualitative or quantitative PCR	98,182	459 (0.5%)	NCDC provided	HCV RNA by point of care test (GeneXpert*) or laboratory-based PCR
Harm reduction providers	Rapid tests were used to test for anti-HCV, and on-site HCV RNA testing was done using a GeneXpert machine. People tested in this model were generally beneficiaries of the harm reduction service (needle and syringe provision or other services) or their family members	5,204	469 (9.0%)	Privately purchased	HCV RNA by point of care test (GeneXpert*)
Specialized provider	Anti-HCV testing was performed at specialized HCV treatment facilities, including clinics with a focus on infectious diseases. Some clinics sent anti-HCV positive samples to the Lugar Center for viremia testing, while others performed viremia testing on site using qualitative or quantitative PCR. Since 2018, the majority of tests have been sent to the Lugar Center for viremia coreAg testing	92,116	4,889 (5.3%)	NCDC provided	HCV coreAg

Abbreviations: HCV, hepatitis C virus, NCDC, National Center for Disease Control and Public Health, anti-HCV, hepatitis C virus antibody; *the point of care HCV RNA testing system used is GeneXpert

From the launch of the decentralization of hepatitis C care and treatment among PHC and HRP in August 2018–2022, a total of 11 PHC and 6 HRP settings throughout the country were providing hepatitis C screening and care services. In the initial phase of decentralization by the government, only HCV treatment-naïve patients with no or mild fibrosis (FIB-4 score <1.45) were treated using simplified diagnostics and treatment monitoring in PHC and HRP sites. Treatment eligibility criteria were later expanded to those with FIB-4 scores between 1.45 and 3.25, while persons with cirrhosis were referred to specialized clinics. Decentralized HRP sites had elastography on site, but PHC referred to specialized sites for elastography if needed.

Overall, more than 97% of treated patients diagnosed through the hospital models or specialized provider were treated by specialized providers. Of those diagnosed in PHC, 35.9% were treated at PHC and the remainder at specialized providers. Of those diagnosed in HRP, 60.8% were treated at HRP, 0.6% at PHC, and the remainder at specialized providers. We used a cut-off date of 31 December 2022 for treatment initiation with aggregated data exported on 18 September 2023.

### Costs

Cost data exported on 9 May 2022 were provided by the Ministry of Internally Displaced Persons from the Occupied Territories, Labour, Health, and Social Affairs of Georgia (hereafter referred to as MoH), National Center for Disease Control and Public Health (NCDC) and service providers. All data were anonymised; authors outside of NCDC did not have access to identifiable data. From these data we calculated unit costs for anti-HCV tests, viremia tests (HCV core antigen or HCV RNA), patient-level totals for treatment and monitoring, and cost of care for liver disease. Costs were collected in Georgian Lari (GEL), inflated to 2022 values using the World Bank consumer price index for Georgia, and converted to USD using an average exchange rate for 2022 of 2.915 GEL/USD. Costs are presented from the health system perspective, including both provider (MoH) and patient costs, accounting for patient co-payments where applicable, and the value of donated drugs. The cost of anti-HCV screening tests was based on unit cost of tests, assuming testing was provided within existing services, viremia test costs were based on reimbursement records and laboratory data, while treatment costs were based on MoH reimbursement to service providers at the patient level. Full details of costing methodology for anti-HCV screening tests, viremia tests, treatment and monitoring, DAA costs, and liver disease care are provided in the Section A in S1 Appendix.

### Decision tree model

Each patient was assigned to one of the five screening strategies described above based on their first anti-HCV positive screening result recorded in the elimination program, or most recent negative if no positive results. To account for potential duplicate entries, repeated test results for the same person within 3 days of a previous test were excluded.

**Fig 1:**
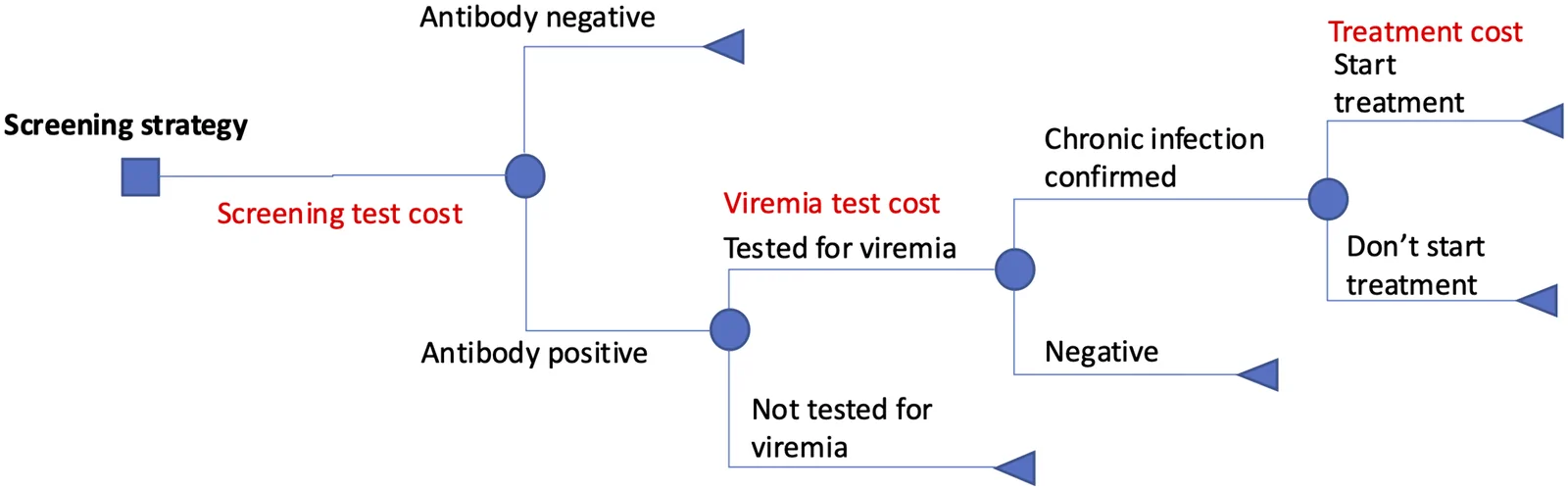
Decision tree model of the hepatitis C diagnostic pathway, used to calculate cost per person treated under each screening pathway.

For each screening strategy in each year during 2018–2022, the decision tree shown in Fig 1 was used to calculate the total cost per person treated. First, the total diagnosis cost for each year was calculated by multiplying the anti-HCV test cost by number of tests given, added to the viremia test cost multiplied by number of persons tested for viremia within that year. As treatment did not always occur in the same year as screening, and treatment costs changed over time, the treatment cost for each year of screening was calculated as the treatment cost multiplied by the number starting treatment in each year that had been screened in a particular year, and accounting for patient level costs of treatment. Screening numbers by year and when and where they were treated are shown in Table A in S1 Appendix. The sum of diagnostic and treatment costs was then divided by the number starting treatment to get the annual cost per person treated under each screening strategy.

### Dynamic model

We used a previously published and validated deterministic compartmental ordinary differential equation model of HCV transmission in Georgia to project the long term costs and impacts of treatment, including dynamically capturing the impact of treatment on incident infections [7,10]. Briefly, the compartmental model was designed to represent the past and current HCV epidemic in Georgia, accounting for HCV transmission among people who inject drugs (PWID) and the general population. The model is stratified by infection status, sex, age, liver disease status, and injecting drug use. Successful treatment leads to SVR, where individuals return to susceptible compartment and are able to be re-infected, with transmission rate different between PWID and the rest of the population. The model was parameterised and calibrated to data from the 2015 and 2021 national sero-surveys and PWID surveys as described previously [7, 10]. For consistency across strategies the model accounts for observed treatment numbers through the end of 2022, with the impact of treatment projected forward for 20 years. For this analysis, the model was adapted and run in Matlab 2023b, with output files analysed in R version 4.4 [11]. We use the set of 76 parameters under the filtered scenario which fit the results of the 2021 serosurvey as described in a previous publication [7].

### Health-related quality of life

We assigned health-related quality of life utility weights to each modelled liver disease state (mild, moderate, cirrhosis, decompensated cirrhosis, or hepatocellular carcinoma (HCC)) [8,12]. The utility weights and uncertainty ranges were, for mild disease 0.83 (0.81–0.85), for moderate disease 0.81 (0.77–0.85), cirrhosis 0.74 (0.68–0.8), decompensated cirrhosis 0.66 (0.46–0.86), and HCC 0.65 (0.44–0.86).

### Cost-effectiveness analysis

A total of 80,897 patients were treated by the end of 2022, with 38,522 treated during 2018–2022. The number of treated patients identified from each of the screening strategies during 2018–2022 were: 4233 for the centralized hospital sector strategy, 5632 for the decentralized hospital sector strategy, 459 for the PHC strategy, 469 for the HRP strategy, and 4889 for the specialized provider strategy. To estimate the impact in terms of QALYs gained due to each screening strategy, we compared the impact of the full set of treatments through 2022 compared to removing the treatments from each of the screening strategies. This reflects the impact of each screening strategy in the context where multiple strategies are in place at the same time, each with a marginal contribution to the overall treatment coverage. For simplicity of comparison and to capture the long-term impacts of the treatments during 2018–2022, HCV treatment is modelled to stop after 2022.

We undertook a probabilistic sensitivity analysis with 500 samples from each parameter. First, we re-sampled 500 values for the number in each liver disease state by year from the dynamic model outputs [7]. QALY weights were sampled from triangular distributions. Total treatment costs per patient (including the cost of case finding from each screening pathway) were sampled from a normal distribution assuming a standard error of 5% of the mean treatment cost for each pathway in each year. Cost of liver disease care was also sampled from a normal distribution using a standard error of 5% of the mean value. The decision tree parameters (numbers diagnosed and treated under each screening pathway) were assumed to be fixed.

The mean difference in total costs and total QALYs by pathway through 2040 was calculated in comparison to the baseline to obtain the incremental cost-effectiveness ratio (ICER). The ICER is presented in terms of cost per QALY from the health system perspective with a 3% annual discount rate for both costs and QALYs. We also conduct a fully incremental analysis to identify dominant screening strategies.

We conducted sensitivity analyses on the perspective by (1) excluding donated DAA costs (provider and patient perspective); (2) excluding cost of liver disease and DAAs to show the impact of the program without accounting for averted healthcare costs (elimination program perspective); and (3) we applied 2022 costs for each pathway for all years during 2018–2022, including DAA costs, to represent health system perspective in which a treatment program is started from scratch with lower drug costs, to inform policy in other settings. In addition, we conducted a standard sensitivity analysis on the base case health system perspective with 0% discount rate or a shortened time horizon to 2030.

### Ethics

This study protocol was reviewed by the Institutional Review Board (IRB) of the Georgian National Center for Disease Control and Public Health (IRB # 2020–060; amendment IRB # 2022–026) and deemed exempt from full IRB review.

## Results

### Unit costs

Unit costs for antibody screening, viremia testing, and treatment within each screening pathway and year are presented in Table B in S1 Appendix. Treatment costs decreased over time, with a clear shift from 2019 to 2020, due primarily to a reduction in the cost of DAAs. Drug costs then remained relatively stable over 2021–2022, between $227 and $252 per course of treatment in 2022. The non-drug component of treatment costs decreased consistently across all treatment pathways each year. In 2018, HRP settings had the lowest total treatment cost (including DAAs and treatment monitoring) of $3942 while specialized providers had the highest at $4247. In 2022, the HRP pathway still had the lowest treatment cost at $300 while the highest cost was for specialized providers at $338.

A total of 886 persons in 2015 and 770 persons in 2016 were included in the financial database for liver disease care; 273 people had costs recorded in both years. The cost per person treated per year was $280.58 for moderate liver disease, $420.08 for compensated cirrhosis, and $830.49 for decompensated cirrhosis, which we assumed applied to HCC.

### Screening strategies

The number of patients included in each screening pathway and the cascade of care for hepatitis C through treatment outcome are shown in Table 2. Hospital-based screening reached the most patients for anti-HCV testing, with both the centralized and decentralized strategies conducting approximately 1 million anti-HCV tests over 2018–2022, compared to fewer than 120,000 tests in PHC and specialized provider sites, and 5,505 tests in HRP. However, hospital-based testing was more likely to be repeated, with 1.57 tests per unique person in centralized and 1.38 tests per person in decentralized strategies, compared to 1.06–1.29 in the other strategies. HRP, followed by specialized providers, had the highest anti-HCV positivity rate among persons tested, with 17.7% and 8.8% respectively, compared to less than 3% in the other settings. These two strategies also had higher rates of viremia testing among those that were anti-HCV positive compared to hospital and primary healthcare. The proportion of anti-HCV positive patients with chronic HCV infection was also lower in hospital settings, which could be due to re-testing of patients who had already been treated. Specialized providers were the most likely to link diagnosed patients to the treatment program (87.9%), followed by HRP (77.7%) and PHC (73.6%), with much lower rates of linkage to care among persons diagnosed in hospital settings (55.0–64.7%). However, once patients were enrolled in the hepatitis C treatment program, rates of starting (93–95%) and completing (90–94%) treatment and of achieving SVR (97–99%) were similar across all strategies.

**Table 2:** Cascades of care for each hepatitis C screening pathway in Georgia. The number of persons at each step in the care cascade with percent who completed each step compared to the number in the previous row.

	Hospital Centralized (2018–2022)		Hospital Decentralized		Primary Healthcare		Harm reduction providers		Specialized Provider (2018–2022)	
Tests Conducted*	1,047,456		969,697		119,186		5,505		118,460	
**Unique Persons Screened**	667,124	1.57 TPP	700,212	1.38 TPP	98,182	1.21 TPP	5,204	1.06 TPP	92,116	1.29 TPP
Anti-HCV Positive*	15,469	2.3%	18,146	2.6%	1,173	1.2%	923	17.7%	8,123	8.8%
Tested for Viremia*	11,734	75.9%	14,434	79.5%	811	69.1%	812	88.0%	7,494	92.3%
Chronic HCV* Infection	8,192	69.8%	9,338	64.7%	648	79.9%	649	79.9%	5,844	78.0%
**Enrolled in Program**	4,502	55.0%	6,040	64.7%	477	73.6%	504	77.7%	5,134	87.9%
Started Treatment*	4,233	94.0%	5,632	93.2%	459	96.2%	469	93.1%	4,889	95.2%
**Completed Treatment**	3,895	92.0%	5,184	92.0%	430	93.7%	422	90.0%	4,558	93.2%
**Eligible for SVR**	3,750	96.3%	4,891	94.3%	419	97.4%	403	95.5%	4,413	96.8%
**Tested for SVR**	2,823	75.3%	3,279	67.0%	323	77.1%	300	74.4%	3,177	72.0%
**SVR Achieved**	2,785	98.7%	3,238	98.7%	313	96.9%	292	97.3%	3,115	98.0%

Abbreviations: TPP, tests per person; HCV, hepatitis C virus, anti-HCV, HCV antibody; SVR, sustained virologic response. *indicates steps represented in the decision tree model (Fig 1).

### Cost per diagnosis and treatment

**Fig 2:**
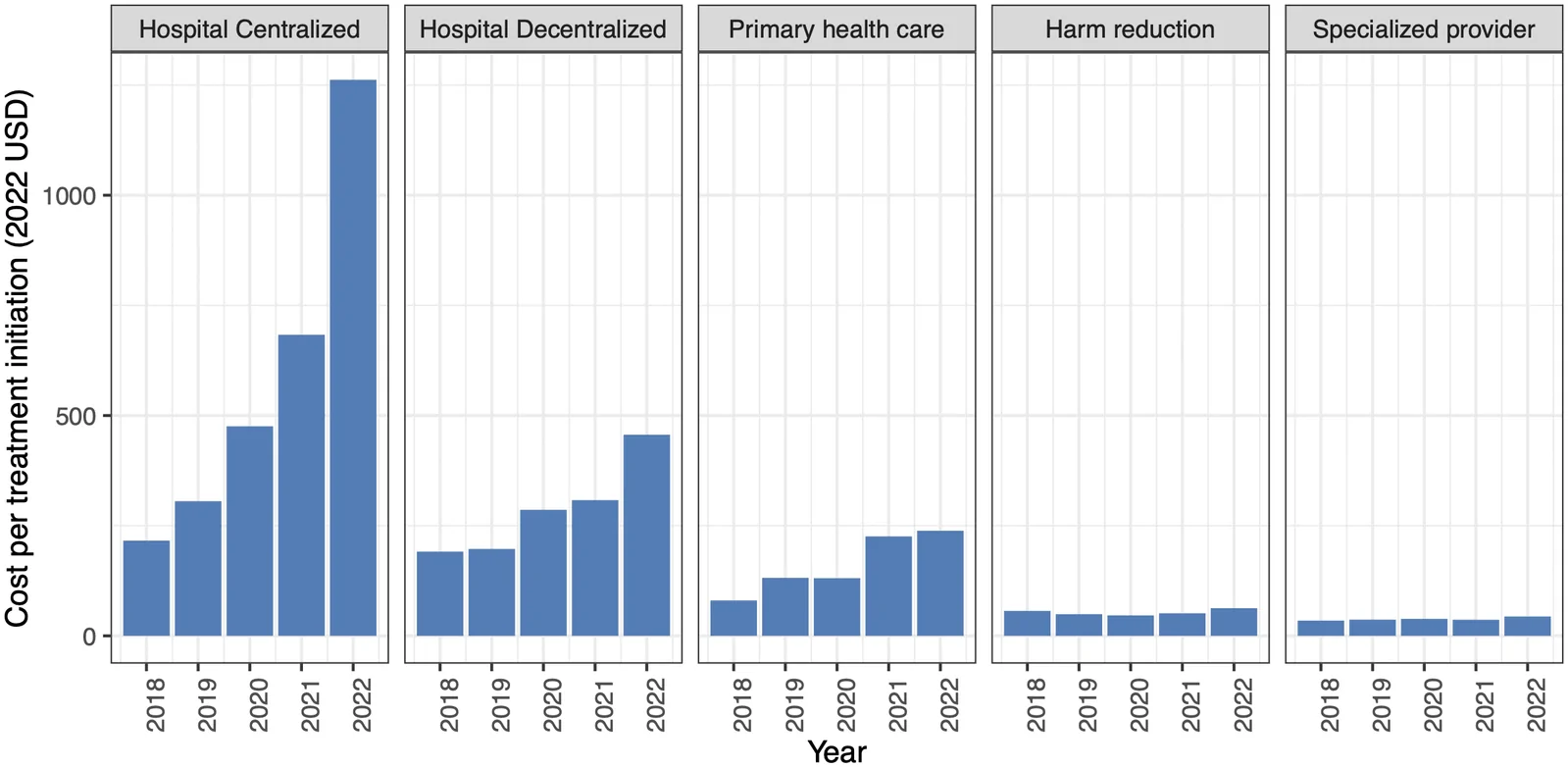
Cost of hepatitis C diagnosis per treatment initiation; for each person treated by screening pathway and year under the hepatitis C elimination program in Georgia, 2018–2022.

Case finding costs are driven by changes in yield of positive test results, which decrease over time in hospital settings. The diagnostic costs (including anti-HCV and viremia testing) per treatment initiation increased from $216 in 2018 to $1262 in 2022 in the centralized hospital strategy, and from $191 to $456 over the same period in the decentralized hospital strategy. PHC costs also increased from $80 to $239, while HRP and specialized provider diagnosis costs were relatively stable and between $34 to $63 per patient treated (Fig 2).

**Fig 3:**
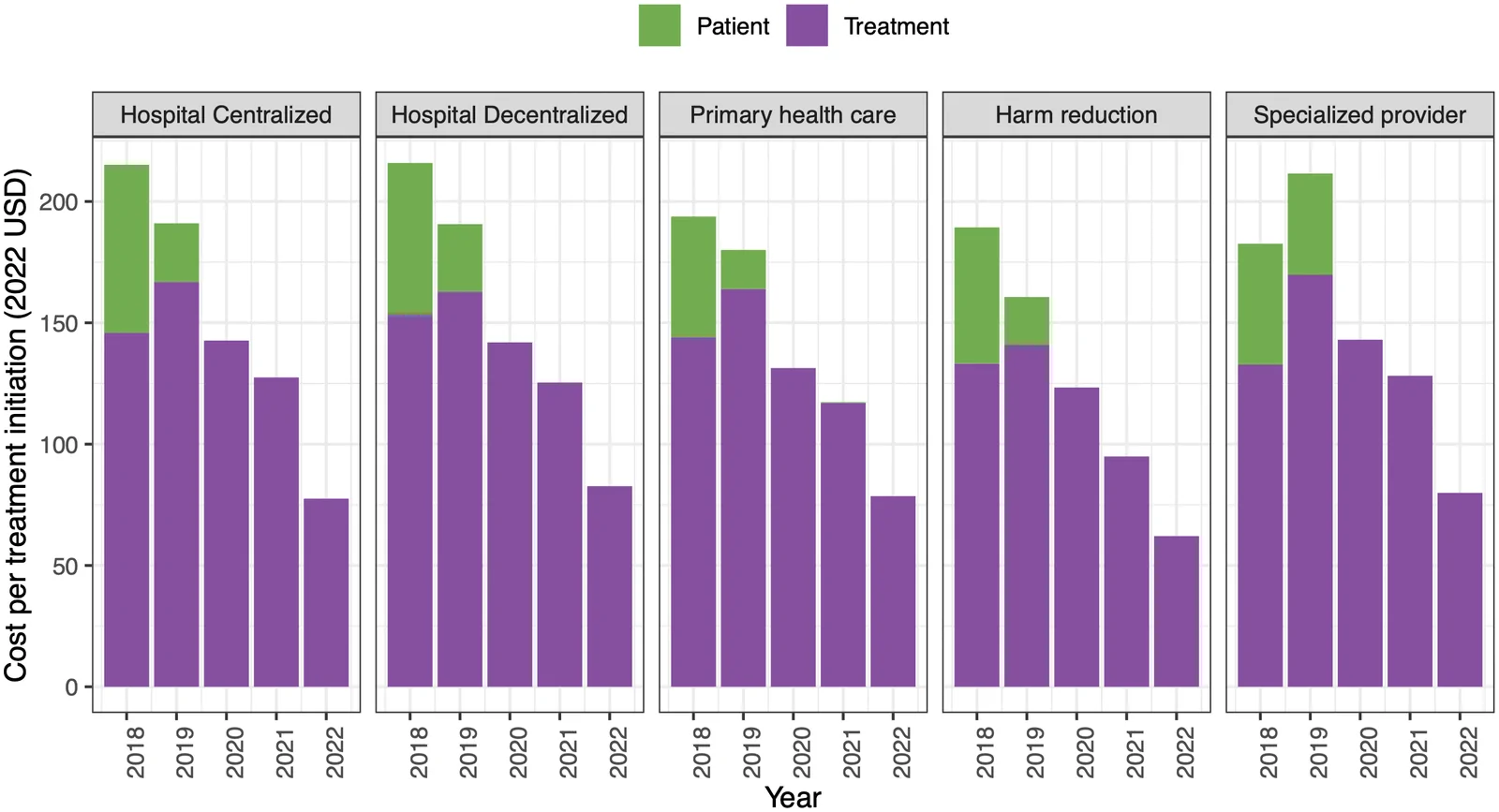
Cost of hepatitis C treatment monitoring; for each person treated by screening pathway and year under the hepatitis C elimination program in Georgia, 2018–2022. Green bars are patient out of pocket costs during treatment, purple bars are costs to elimination program during treatment. DAAs: direct-acting antivirals.

**Fig 4:**
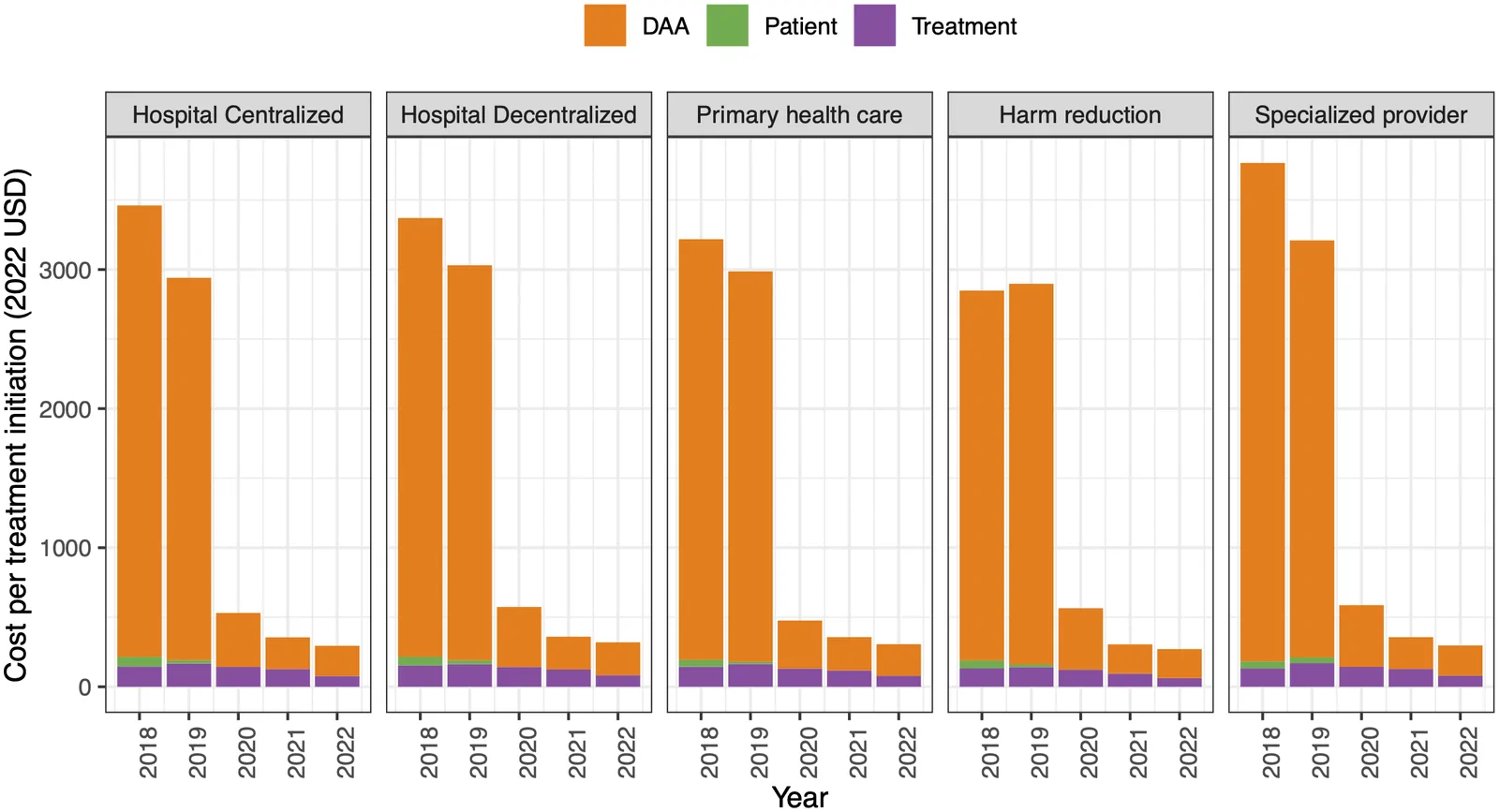
Cost of DAAs with cost of hepatitis C treatment monitoring, for each person treated by screening pathway and year under the hepatitis C elimination program in Georgia, 2018–2022. Green bars are patient out of pocket costs during treatment, purple bars are costs to elimination program during treatment, orange bars DAA costs. DAAs: direct-acting antivirals.

The total costs per treatment initiation based on treatment monitoring alone within each strategy and excluding diagnostic costs also decreased over time in all settings, with costs fairly similar across pathways (Fig 3). When DAA costs are accounted for, there is a large decrease in treatment cost from 2019 to 2020 due to the switch to generic regimens followed by a gradual decrease reaching $300–$338 per treatment initiation in 2022 (Fig 4). The cost of treatment accounts for actual year of treatment initiation (for example, 50% of those screened in 2018 were treated in 2018) which results in the cost per treatment initiation by pathway being lower than the unit costs by year in some cases (Tables A and B in S1 Appendix).

Overall, the cost of DAA drugs has changed from being the primary driver of treatment costs to being similar or less than the cost of case finding diagnostics (Fig 2, Fig 3, Fig 4). Across all years, HRP had the lowest cost per treatment initiation, due to high yield and slightly lower treatment costs (Table 3). Based on more recent 2022 costs applied to the cascade of care and including DAA costs, the cost per treatment initiation is lowest in HRP ($350) followed by specialized providers ($375), with PHC $423, hospital decentralized and hospital centralized the highest at $589 and $681, respectively, per patient treated.

**Table 3:** Total cost per hepatitis C treatment initiation in Georgia, by screening pathway and year of screening, in 2022 USD, and total QALY losses averted by treatments under each screening pathway.

Year	Hospital Centralized	Hospital Decentralized	Primary Healthcare	Harm reduction providers	Specialized Provider
Total cost including DAAs (health system perspective)					
2018	3617	3480	3228	3179	3844
2019	3126	3057	3008	2821	3080
2020	1602	1399	1116	967	1294
2021	1052	683	588	388	412
2022	1591	786	544	363	381
Average over 2018–2022	1861	1319	1491	1012	1512
Average over 2018–2022 using 2022 costs	681	589	423	350	375
Excluding DAA costs (provider and patient perspective)					
2018	425	393	270	231	261
2019	490	388	311	211	236
2020	623	435	271	171	195
2021	806	434	345	152	166
2022	1349	545	315	135	130
Overall	280	221	157	97	118
Overall using 2022 costs	438	348	193	122	123
QALY losses averted through 2040 by treatments under base case assumptions					
	1975	2310	2257	163	187

Abbreviations: QALY, quality adjusted life years; DAA, direct-acting antiviral

### Cost-effectiveness

ICERs and 95% credible intervals are shown in Table 4 and the cost-effectiveness plane in Fig A in S1 Appendix, and detailed ICER tables in Table C in S1 Appendix (compared to baseline) and Table D in S1 Appendix (fully incremental analysis). Under the base case scenario over a time horizon to the end of 2040, and including liver disease costs and the cost of DAA drugs, none of the pathways are cost effective at the WTP threshold of $1,337/QALY but are likely to be cost-effective at a higher threshold of 1 GDP per capita ($6,685) (Table 4). Under this scenario, in a fully incremental analysis specialized provider and hospital centralized are dominated and PHC is extendedly dominated, with HRP and hospital decentralized cost-effective at the higher WTP threshold (Table D in S1 Appendix). However, when DAA costs are removed (provider plus patient perspective), all screening pathways are cost effective compared to baseline at the lower threshold, with specialized provider, HRP, and PHC strategies being cost saving due to higher screening yields. Using 2022 costs for the whole care pathway, and including DAA costs, the result is similar, all ICERs compared to baseline are below the WTP threshold with the mean ICER for specialized providers, HRP, and PHC all cost-saving. In both of these lower costs scenarios, in the fully incremental analysis, specialized provider is the most impactful in terms of costs saved and QALYs gained, while no intervention and centralized treatment are both dominated. Excluding liver disease costs as well as DAAs results in the mean ICER for all scenarios remaining cost-effective at the lower threshold, with 76.6%, 86.0%, and 99.8% of model runs cost-effective for centralized, decentralized, and PHC strategies, respectively, and all model runs cost-effective for specialized providers and HR strategies. A shorter time horizon to 2030 means the strategies are no longer cost-effective due to the up-front cost of the treatments and longer time horizon for QALY gains. Without discounting, all ICERs are lower compared to the base case, falling above 20% GDP per capita but below 1 GDP per capita.

**Table 4:** Mean incremental cost-effectiveness ratio (ICER) in terms of cost per quality adjusted life year (QALY) in 2022 US dollars with a time horizon to 2040, and 95% credible interval for each hepatitis C screening pathway compared to baseline, for base case model assumptions and scenario analyses.

Pathway	Base case (health system perspective)	Without DAA costs (provider and patient perspective)	No liver disease or DAA costs (elimination program perspective)	2022 costs (health system perspective for new treatment program)	Shorten time horizon to 2030	0% discounting
Hospital Centralized	5752 (4014, 8384)	298 (13, 665)	1179 (833, 1704)	630 (332, 1155)	18454 (12573, 27686)	3954 (2754, 5759)
Hospital Decentralized	4258 (2950, 6333)	59 (-252, 404)	1068 (751, 1579)	476 (146, 937)	15496 (10492, 23075)	2808 (1926, 4213)
Specialized provider	4948 (3415, 7402)	-422 (-791, -122)	499 (355, 733)	-84 (-393, 224)	16110 (10897, 24543)	3364 (2313, 5066)
Harm reduction	3464 (2161, 5346)	-826 (-1354, -391)	548 (367, 880)	-323 (-701, 69)	14178 (9141, 22540)	2134 (1245, 3397)
Primary healthcare	4870 (3141, 7555)	-374 (-739, -26)	767 (511, 1175)	-42 (-379, 304)	17219 (11402, 26868)	3226 (2007, 5081)

Abbreviations: ICER, incremental cost-effectiveness ratio; QALY, quality adjusted life years; DAA, direct-acting antiviral

## Discussion

Our results highlight significant reductions in the cost of HCV treatment during 2018–2022 across various diagnostic strategies in Georgia, driven by changes in screening yield, the introduction of generic drugs, and simplification of treatment protocols. We find that from the provider and patient perspective, with DAA drugs donated to the program throughout, the elimination program across all evaluated pathways is likely to have been cost-effective, and may have been cost-saving for screening pathways outside of hospitals due to averted liver disease care costs. The same program implemented based on 2022 costs including DAA costs would similarly be cost-effective or cost-saving, indicating that DAA costs are less of a barrier to hepatitis C elimination than they were when elimination targets were introduced in 2016 [13,14]. This result illustrates that HCV elimination strategies will be even more cost-effective with today’s generic DAA prices and streamlined diagnostic approaches, making Georgia’s experience informative for policymakers in other settings.

We identified clear differences in the cost-effectiveness of different screening pathways, with screening in HRPs and PHCs, as well as specialized providers leading to higher yield, with better linkage to care and treatment uptake. In Georgia, HCV screening is mandatory for all hospital inpatients, and early in the elimination program this allowed identification of persons with HCV infection without previously identified risks of HCV infection. However, over time the screening yield and efficiency of the diagnostic pathway decreased in hospital settings, while it was maintained by the providers who use a more targeted approach for screening. This change over time emphasizes the need for different approaches as fewer persons remain to be diagnosed and linked to care.

### Strengths and limitations

A key strength of our study is the use of comprehensive, real-world data on costs and care cascades. We also build on robust modelling techniques that provide a detailed analysis of costs and outcomes. However, real-world data introduced some challenges and limitations around developing the care cascade and we had limited data to support some assumptions. Patient pathways were complex, with many patients being screened in one setting but treated in another, or with a long delay between diagnosis and treatment. We were able to capture this within our cost estimates by focusing our analysis on cohorts defined by their screening site (where they had their first positive test) and accounting for costs of treatment for persons wherever and whenever they were treated. Although this loses nuance in the cascade of care that may depend on where patients enroll in the program or decide to be treated, it accounts for the reality of when and where people were treated. In addition, the total number of people reached through harm reduction is an underestimate, as some patients are tested anonymously in these settings and therefore were not linkable across the care cascade [4].

Some costs also had to be simplified, and we assumed a normal distribution for costs. We did not estimate the staff time or overhead associated with screening tests as no data were available on this; therefore we assume that screening occurs within existing services in this evaluation, focusing on the incremental cost to hospital, primary care, and harm reduction services of adding HCV testing to existing visits. For HCV RNA costs, test type was not specified in the financial database, so we had to assume all PCR tests, whether point of care (GeneXpert) or Qualitative laboratory-based tests, had the same cost. We were able to triangulate HCV core Antigen (coreAg) test numbers with laboratory records to account for required PCR re-testing of “grey zone” results in which the coreAg test is inconclusive. For the cost-effectiveness analysis, the impact of treatment was evaluated compared to the full treatment numbers achieved in the elimination program; that is, we removed the number of treatments achieved from each strategy to calculate the incremental benefits of those treatments. Evaluating the impact of the treatment numbers achieved by a particular strategy as the only treatments modelled (in the absence of other treatments given) would likely give a different result with higher impact per treatment. Finally, we acknowledge that as the screening strategies target different populations, they are likely to combined in a suite of screening approaches accounting for equity, feasibility, effectiveness, as well as cost-effectiveness. Our results aim to provide insight on how cost-effectiveness of the strategies may differ in relation to the cascade of care and unit costs.

### Comparison to other literature

Cost-effectiveness of HCV screening approaches for elimination have been evaluated in other settings around the world, including high income (Ireland [15], Canada/USA [16], Spain [17], Italy [18], Germany [19], South Korea [20], England [21], Netherlands [22]), upper-middle income (China [23], Iraq [24]), and lower-middle income (Pakistan [25], Cameroon/Cote d’Ivoire/Senegal [26], Egypt [27]) countries. Other than the evaluation of the national programme in Egypt which was retrospective, studies generally present hypothetical scenarios either focused on diagnostic approaches or on which populations to target for screening, and consider the impact of starting large scale screening or treatment programs, while some studies evaluate programmes focused on key populations only. The study in China is the only one to consider how optimal targeted screening approaches will change as hepatitis C prevalence decreases [23]. As Georgia implemented a national hepatitis C elimination program earlier than many other countries, our assessment of the real-life differences between screening strategies should prove useful to other countries as they develop strategies.

Previous studies have evaluated components of the cost-effectiveness of screening and treatment for hepatitis C. Two studies compared the cost-effectiveness of alternative diagnostic strategies where HCV coreAg or HCV RNA is used for the viremia test. Sadeghimehr et al found that antibody followed by HCV coreAg testing was the cheapest option and likely the most cost-effective option, though using antibody followed by HCV RNA instead led to more QALYs gained [28]. Their unit costs were gathered from literature and experts within Georgia’s hepatitis C elimination program and their model considered disease progression within persons with HCV. Adee et al also modelled disease progression with a Markov model, but diagnostic costs were based on a trial and healthcare costs were adapted from USA data; the most cost-effective approach used on-site RNA tests and Fibroscan for liver disease staging [29]. Georgia was one country included in a model of the cost-effectiveness (in terms of cost per person cured) of introducing HCV self-testing in addition to standard of care [30]. Therefore this study, which retrospectively evaluates the real-life implementation of different screening approaches within Georgia’s elimination program, and projects long term outcomes within a dynamic model, provides a more complete picture to inform Georgia’s policies as they reach the later stages of elimination. In comparison to our previous paper evaluating the first stages of HCV treatment within the program, [8] this study demonstrates how the effectiveness and cost-effectiveness of case-finding approaches will change as the number of people already treated increases, but similarly emphasises that the elimination program would not be cost effective while accounting for the full cost of DAAs prior to the drastic decrease in DAA costs seen in recent years.

### Implications

Our study supports the strategic use of a variety of screening and treatment strategies as cost-effective approaches for hepatitis C elimination in Georgia. Although multiple approaches will be needed to reach different groups, effectiveness will change over time. Despite drastic reductions in the cost of treatment, the reduction in yield from mass screening diminishes, thus reducing its cost effectiveness compared to more targeted approaches. This observation is crucial for policymakers, as it provides evidence that a pivot towards more targeted screening strategies may be necessary in mid to late stages of hepatitis C elimination programs. Furthermore, comparative cost-effectiveness of screening and treatment approaches in Georgia may be particularly relevant for other countries in the Eastern Europe and Central Asia region, where financial constraints and high hepatitis C prevalence often coexist, emphasizing the importance of using efficient care pathways alongside access to affordable DAAs.

## Supporting information

S1 Appendix S1 Appendix contains: Section A: Supplementary Methods, Section B: CHEERS checklist; Supplementary Tables A, B, C, D, and Supplementary Figs A, B. (PDF)
