# Supplementary material for: Cost and cost-effectiveness of alternative screening and diagnostic pathways for achieving hepatitis C elimination in the country of Georgia
Source: PLOS Glob Public Health. 2026 Sep 22;6(9):e0007122. doi: 10.1371/journal.pgph.0007122 (PMC13596791; doi:10.1371/journal.pgph.0007122)
Supplement: Supplementary file 1 [file pgph.0007122.s001.pdf]

S1 Appendix

Cost and cost-effectiveness of alternative screening and diagnostic pathways for achieving hepatitis C elimination in the country of Georgia

Josephine G. Walker, Irina Tskhomelidze Schumacher, Shaun Shadaker, Tamar Gabunia, Rania A. Tohme, Akaki Abutidze, Vladimer Getia, Peter Vickerman

Table of Contents

Section A: Supplementary Methods ***2***

Costing methodology **2**

Section B: CHEERS 2022 Checklist ***4***

Table A ***7***

Table B ***11***

Table C ***12***

Table D ***14***

Fig A ***15***

Fig B ***16***

Section A: Supplementary Methods

Costing methodology

Screening tests

NCDC provided tests to primary health care and specialized HCV provider sites, as well as other public screening sites not included in this analysis. All other settings purchased tests privately. The unit cost of tests provided by NCDC was calculated from expenditure lists of screening supply data from 2018-2020, and the cost in 2021 and 2022 was assumed to be the same as in 2020. Screening supplies included Hepatitis C rapid tests and consumables (gloves, alcohol wipes, cotton, absorbent paper, disposable safety needles, sharps waste containers, biosecurity bags, disposable gowns, markers). We accounted for the entire amount spent on materials each year, then determined the cost per rapid test by dividing the yearly spending by the quantity of screening tests bought that year.

The cost of the screening tests acquired by private purchase was gathered by interviewing service providers and taking the midpoint of reported purchase price. Reported values were assumed to be in 2022 GEL and the same unit cost was used for each year. Privately purchased tests were generally 4 in 1 tests that screen for HIV, HBV, and syphilis as well as HCV antibodies.

We assumed all screening sites where tests were provided by NCDC had the test cost as calculated from NCDC data and all other sites used the range provided for private purchase. Screening costs did not account for overheads or staff time, tests were generally provided within existing services and we were not able to access data on staff time.

Viremia Diagnostics

Tests used for confirmation of active HCV infection included HCV core antigen (HCV coreAg) or PCR for HCV RNA (qualitative PCR or GeneXpert). For GeneXpert and Qualitative PCR we used the cost provided from the reimbursement database, the price for qualitative PCR and GeneXpert was fixed at 60 GEL. Where test reimbursement amounts remained constant over 2018-2022, accounting for inflation results in effective test costs reducing each year (for example 60 GEL in 2018 would be 65.44 GEL in 2022 values, but the reimbursed amount remained 60 GEL across the time period).

HCV coreAg was approved as an alternative assay for chronic HCV infection at the Lugar Center in December 2017. Materials for HCV coreAg testing were procured centrally. To calculate the unit cost per test we used the NCDC procurement database, with all relevant costs summed for each year available (2018-2021), and then divided by the number of HCV coreAg tests conducted per year. To account for 32.7% of HCV coreAg tests having a grey zone result requiring re-testing with PCR we added 19.6 GEL per HCV coreAg test.

Treatment and monitoring

To calculate the cost of treatment, including pre-treatment assessment, genotyping (prior to 2020 when pan-genotypic treatment was introduced), treatment monitoring, DAA and other drug costs, and SVR assessment, we matched patient-level data from the treatment database to financial reimbursement records, using anonymised unique identifiers. We received costs

for treatment and monitoring from the MoH covering financial reimbursement related to the HCV elimination program from 2015 through July 2022. Payment codes were classified according to stage of treatment pathway and summed separately to account for patient co-payments (prior to 2019) and payments by MoH. Patients were classified according to their screening pathway and total costs summed at the individual level, and then averaged across all patients classified within each pathway by year of treatment. In this way, true costs for the patients screened in each pathway were identified regardless of where they were treated.

DAA costs

DAAs used in the program were donated by Gilead Sciences so prices for these drugs were calculated based on the current market price of the drugs as defined by the National Forensics Bureau. Non-DAA drugs (ribavirin and interferon) costs were based on the average purchase price by MoH. The cost of HCV treatment using each regimen was calculated for each calendar year, with that year's unit cost per box multiplied to cover the treatment length (normally 12 or 24 weeks). Regimens with ribavirin used weight-based dosing, we assumed that all patients would weigh ≥75 kg on average. Costs were then assigned to each patient based on their reported treatment regimen.

Cost of liver disease

Patients with advanced liver disease caused by active HCV infection received inpatient care covered by a state vertical program on management of infectious diseases. To evaluate the long term cost of liver disease within the model, we extracted annual inpatient costs defined as treatment for chronic viral hepatitis, either with highly active pathologic process (assumed to refer to moderate liver disease), with cirrhosis (assumed to refer to compensated cirrhosis), or with cirrhosis, ascites and/or encephalopathy, and/or hepato-renal syndrome (assumed to refer to decompensated cirrhosis, and assuming the same cost applies to HCC due to lack of data on liver cancer costs). Data were only available for 2015 and 2016, total costs in each group per year were inflated and converted to 2022 USD using the same method as for the other costs, then divided by the number of unique patients reached to estimate the cost per patient per year. In the base case we assume all patients with liver disease incur these costs.

Section B: CHEERS 2022 Checklist

Topic	No.	Item	Location where item is reported
**Title**			
	1	Identify the study as an economic evaluation and specify the interventions being compared.	Title
**Abstract**			
	2	Provide a structured summary that highlights context, key methods, results, and alternative analyses.	Page 1, Abstract
**Introduction**			
**Background and objectives**	3	Give the context for the study, the study question, and its practical relevance for decision making in policy or practice.	Page 2, Introduction
**Methods**			
**Health economic analysis plan**	4	Indicate whether a health economic analysis plan was developed and where available.	Not done, not reported
**Study population**	5	Describe characteristics of the study population (such as age range, demographics, socioeconomic, or clinical characteristics).	Page 5, Cost-effectiveness analysis section
**Setting and location**	6	Provide relevant contextual information that may influence findings.	Page 3, Screening strategies section
**Comparators**	7	Describe the interventions or strategies being compared and why chosen.	Page 5, Cost-effectiveness analysis section
**Perspective**	8	State the perspective(s) adopted by the study and why chosen.	Page 4, Costs section
**Time horizon**	9	State the time horizon for the study and why appropriate.	Page 6, Cost-effectiveness analysis section
**Discount rate**	10	Report the discount rate(s) and reason chosen.	Page 6, Cost-effectiveness analysis section
**Selection of outcomes**	11	Describe what outcomes were used as the measure(s) of benefit(s) and harm(s).	Page 6, Cost-effectiveness analysis section
**Measurement of outcomes**	12	Describe how outcomes used to capture benefit(s) and harm(s) were measured.	Page 4-5, Decision tree model; Dynamic model sections

Topic	No.	Item	Location where item is reported
**Valuation of outcomes**	13	Describe the population and methods used to measure and value outcomes.	Page 5, Health-related quality of life section
**Measurement and valuation of resources and costs**	14	Describe how costs were valued.	Page 4, Costs section
**Currency, price date, and conversion**	15	Report the dates of the estimated resource quantities and unit costs, plus the currency and year of conversion.	Page 4, Costs section
**Rationale and description of model**	16	If modelling is used, describe in detail and why used. Report if the model is publicly available and where it can be accessed.	Page 4-5, Decision tree model; Dynamic model sections
**Analytics and assumptions**	17	Describe any methods for analysing or statistically transforming data, any extrapolation methods, and approaches for validating any model used.	Page 5, Dynamic model section
**Characterising heterogeneity**	18	Describe any methods used for estimating how the results of the study vary for subgroups.	Page 3, Screening strategies section
**Characterising distributional effects**	19	Describe how impacts are distributed across different individuals or adjustments made to reflect priority populations.	N/A
**Characterising uncertainty**	20	Describe methods to characterise any sources of uncertainty in the analysis.	Page 5-6, Cost-effectiveness analysis section
**Approach to engagement with patients and others affected by the study**	21	Describe any approaches to engage patients or service recipients, the general public, communities, or stakeholders (such as clinicians or payers) in the design of the study.	N/A
**Results**			
**Study parameters**	22	Report all analytic inputs (such as values, ranges, references) including uncertainty or distributional assumptions.	Table 2, Table B
**Summary of main results**	23	Report the mean values for the main categories of costs and outcomes of interest and summarise them in the most appropriate overall measure.	Table 3, Table 4
**Effect of uncertainty**	24	Describe how uncertainty about analytic judgments, inputs, or projections affect findings. Report the effect of choice of discount rate and time horizon, if applicable.	Page 9, Cost-effectiveness section

Topic	No.	Item	Location where item is reported
**Effect of engagement with patients and others affected by the study**	25	Report on any difference patient/service recipient, general public, community, or stakeholder involvement made to the approach or findings of the study	N/A
**Discussion**			
**Study findings, limitations, generalisability, and current knowledge**	26	Report key findings, limitations, ethical or equity considerations not captured, and how these could affect patients, policy, or practice.	Discussion section
**Other relevant information**			
**Source of funding**	27	Describe how the study was funded and any role of the funder in the identification, design, conduct, and reporting of the analysis	Page 2, left side, Funding section
**Conflicts of interest**	28	Report authors conflicts of interest according to journal or International Committee of Medical Journal Editors requirements.	Page 2, left side, Competing interests section

From: Husereau D, Drummond M, Augustovski F, et al. Consolidated Health Economic Evaluation Reporting Standards 2022 (CHEERS 2022) Explanation and Elaboration: A Report of the ISPOR CHEERS II Good Practices Task Force. Value Health 2022;25. doi:10.1016/j.jval.2021.10.008

Table A

Hepatitis C Care cascades by screening model and year, including treatment sites and years of treatment in Georgia. The number of persons at each step in the care cascade with percent who completed each step compared to the number in the previous row.

Screening year 2018										
	Hospital Centralized		Hospital Decentralized		PHC		HR		Specialized provider	
**Care Cascade**										
Tests Conducted	320,842		48,579		13,644		336		48,987	
Unique Persons Screened	281,503		47,274		13,229		336		41,862	
Anti-HCV Positive	7,298	2.6%	1,209	2.6%	283	2.1%	48	14.3%	3,236	7.7%
Tested for Viremia	6,026	82.6%	1,055	87.3%	214	75.6%	45	93.8%	2,972	91.8%
Current HCV Infection	4,283	71.1%	734	69.6%	174	81.3%	36	80.0%	2,360	79.4%
Enrolled in Program	2,360	55.1%	452	61.6%	124	71.3%	29	80.6%	2,018	85.5%
Started Treatment	2,255	95.6%	429	94.9%	120	96.8%	25	86.2%	1,962	97.2%
Completed Treatment	2,089	92.6%	401	93.5%	115	95.8%	22	88.0%	1,840	93.8%
Eligible for SVR	2,050	98.1%	395	98.5%	115	100.0%	22	100.0%	1,828	99.3%
Tested for SVR	1,545	75.4%	296	74.9%	82	71.3%	17	77.3%	1,329	72.7%
SVR Achieved	1,517	98.2%	289	97.6%	79	96.3%	17	100.0%	1,301	97.9%
**Treating Site**										
Specialized Provider	2196	97.4%	414	96.5%	85	70.8%	17	68.0%	1946	99.2%
PHC	38	1.7%	4	0.9%	35	29.2%			4	0.2%
HRP	21	0.9%	11	2.6%			8	32.0%	12	0.6%
**Year Treated**										
2018	957	42.4%	35	8.2%	37	30.8%	5	20.0%	1362	69.4%
2019	816	36.2%	314	73.2%	54	45.0%	15	60.0%	403	20.5%
2020	274	12.2%	41	9.6%	18	15.0%	4	16.0%	109	5.6%
2021	86	3.8%	12	2.8%	7	5.8%			46	2.3%
2022	122	5.4%	27	6.3%	4	3.3%	1	4.0%	42	2.1%

Screening year 2019										
**Care Cascade**										
Tests Conducted	218,268		302,320		36,762		2,427		26,479	
Unique Persons Screened	193,293		261,127		35,284		2,381		21,430	
Anti-HCV Positive	3,001	1.6%	6,751	2.6%	473	1.3%	323	13.6%	2,139	10.0%
Tested for Viremia	2,497	83.2%	5,714	84.6%	337	71.2%	283	87.6%	2,033	95.0%
Current HCV Infection	1,789	71.6%	3,818	66.8%	266	78.9%	231	81.6%	1,564	76.9%
Enrolled in Program	1,087	60.8%	2,620	68.6%	192	72.2%	192	83.1%	1,411	90.2%
Started Treatment	1,007	92.6%	2,477	94.5%	185	96.4%	186	96.9%	1,342	95.1%
Completed Treatment	955	94.8%	2,346	94.7%	178	96.2%	180	96.8%	1,298	96.7%
Eligible for SVR	940	98.4%	2,309	98.4%	177	99.4%	179	99.4%	1,291	99.5%
Tested for SVR	709	75.4%	1,554	67.3%	146	82.5%	135	75.4%	947	73.4%
SVR Achieved	704	99.3%	1,538	99.0%	140	95.9%	130	96.3%	930	98.2%
**Treating Site**										
Specialized Provider	980	97.3%	2432	98.2%	111	60.0%	86	46.2%	1334	99.4%
PHC	26	2.6%	22	0.9%	74	40.0%	3	1.6%	2	0.1%
HRP	1	0.1%	23	0.9%			97	52.2%	6	0.4%
**Year Treated**										
2019	608	60.4%	1607	64.9%	127	68.6%	129	69.4%	968	72.1%
2020	298	29.6%	642	25.9%	40	21.6%	46	24.7%	320	23.8%
2021	42	4.2%	110	4.4%	14	7.6%	6	3.2%	29	2.2%
2022	59	5.9%	118	4.8%	4	2.2%	5	2.7%	25	1.9%
**Screening year 2020**										
**Care Cascade**										
Tests Conducted	166,780		220,722		26,256		929		17,198	
Unique Persons Screened	150,457		193,837		24,552		920		14,243	
Anti-HCV Positive	1,843	1.2%	3,934	2.0%	204	0.8%	182	19.8%	1,105	7.8%
Tested for Viremia	1,345	73.0%	3,128	79.5%	148	72.5%	161	88.5%	1,024	92.7%

Current HCV Infection	930	69.1%	1,895	60.6%	113	76.4%	119	73.9%	770	75.2%
Enrolled in Program	503	54.1%	1,237	65.3%	92	81.4%	102	85.7%	696	90.4%
Started Treatment	476	94.6%	1,166	94.3%	87	94.6%	100	98.0%	655	94.1%
Completed Treatment	443	93.1%	1,118	95.9%	83	95.4%	98	98.0%	636	97.1%
Eligible for SVR	422	95.3%	1,074	96.1%	81	97.6%	95	96.9%	631	99.2%
Tested for SVR	323	76.5%	726	67.6%	61	75.3%	73	76.8%	451	71.5%
SVR Achieved	318	98.5%	717	98.8%	60	98.4%	71	97.3%	441	97.8%
**Treating Site**										
Specialized Provider	457	96.0%	1148	98.5%	57	65.5%	33	33.0%	648	99.1%
PHC	15	3.2%	11	0.9%	30	34.5%			4	0.6%
HRP	4	0.8%	7	0.6%			67	67.0%	2	0.3%
**Year Treated**										
2020	302	63.4%	840	72.0%	63	72.4%	77	77.0%	549	83.8%
2021	114	23.9%	209	17.9%	19	21.8%	16	16.0%	84	12.8%
2022	60	12.6%	117	10.0%	5	5.7%	7	7.0%	22	3.4%
**Screening year 2021**										
**Care Cascade**										
Tests Conducted	156,044		196,135		22,401		864		11,812	
Unique Persons Screened	139,546		176,117		21,458		857		11,344	
Anti-HCV Positive	1,685	1.2%	3,299	1.9%	106	0.5%	180	21.0%	847	7.5%
Tested for Viremia	1,123	66.6%	2,589	78.5%	56	52.8%	165	91.7%	774	91.4%
Current HCV Infection	683	60.8%	1,612	62.3%	47	83.9%	142	86.1%	613	79.2%
Enrolled in Program	338	49.5%	1,027	63.7%	37	78.7%	100	70.4%	558	91.0%
Started Treatment	307	90.8%	941	91.6%	36	97.3%	88	88.0%	523	93.7%
Completed Treatment	283	92.2%	888	94.4%	34	94.4%	84	95.5%	492	94.1%
Eligible for SVR	267	94.3%	844	95.0%	34	100.0%	83	98.8%	484	98.4%
Tested for SVR	204	76.4%	553	65.5%	27	79.4%	55	66.3%	342	70.7%
SVR Achieved	204	100.0%	546	98.7%	27	100.0%	54	98.2%	336	98.2%

**Treating Site**										
Specialized Provider	301	98.0%	931	98.9%	20	55.6%	22	25.0%	522	99.8%
PHC	5	1.6%	7	0.7%	16	44.4%			1	0.2%
HRP	1	0.3%	3	0.3%			66	75.0%		
**Year Treated**										
2021	195	63.5%	672	71.4%	29	80.6%	65	73.9%	430	82.2%
2022	112	36.5%	269	28.6%	7	19.4%	23	26.1%	93	17.8%
**Screening year 2022**										
**Care Cascade**										
Tests Conducted	185,522		201,941		20,123		949		13,984	
Unique Persons Screened	165,626		178,598		18,622		937		13,396	
Anti-HCV Positive	1,642	1.0%	2,953	1.7%	107	0.6%	190	20.3%	796	5.9%
Tested for Viremia	743	45.2%	1,948	66.0%	56	52.3%	158	83.2%	691	86.8%
Current HCV Infection	507	68.2%	1,279	65.7%	48	85.7%	121	76.6%	537	77.7%
Enrolled in Program	214	42.2%	704	55.0%	32	66.7%	81	66.9%	451	84.0%
Started Treatment	188	87.9%	619	87.9%	31	96.9%	70	86.4%	407	90.2%
Completed Treatment	125	66.5%	188	30.4%	20	64.5%	38	54.3%	292	71.7%
Eligible for SVR	71	56.8%	269	143.1%	12	60.0%	24	63.2%	179	61.3%
Tested for SVR	42	59.2%	150	55.8%	7	58.3%	20	83.3%	108	60.3%
SVR Achieved	42	100.0%	148	98.7%	7	100.0%	20	100.0%	107	99.1%
**Treating Site**										
Specialized Provider	186	98.9%	617	99.7%	21	67.7%	23	32.9%	405	99.5%
PHC	1	0.5%	2	0.3%	10	32.3%				
HRP	1	0.5%					47	67.1%	2	0.5%
**Year Treated**										
2022	188	100.0%	619	100.0%	31	100.0%	70	100.0%	407	100.0%

Abbreviations: PHC, primary health care; HRP, harm reduction provider; SVR , sustained virological response; HCV, hepatitis C viru

Table B

Mean unit costs for hepatitis C diagnosis and treatment in Georgia per patient by screening pathway and year, in 2022 USD.

Year	Screening pathway	Anti-HCV test cost	Viremia test cost	Treatment monitoring cost	DAA cost
2018	Centralized hospital sector	1.20	16.95	244.32	3963.73
2018	Decentralized hospital sector	1.20	22.45	246.19	3981.69
2018	Primary health care	0.35	22.45	220.75	3811.08
2018	Harm reduction	1.20	22.45	216.63	3725.40
2018	Specialized provider	0.35	16.95	241.70	4005.64
2019	Centralized hospital sector	1.20	18.26	208.22	3638.59
2019	Decentralized hospital sector	1.20	21.96	214.44	3581.87
2019	Primary health care	0.46	21.96	197.61	3556.62
2019	Harm reduction	1.20	21.96	177.85	3400.73
2019	Specialized provider	0.46	18.26	215.91	3511.98
2020	Centralized hospital sector	1.20	19.41	161.65	1401.22
2020	Decentralized hospital sector	1.20	21.89	158.73	1242.85
2020	Primary health care	0.31	21.89	148.51	1075.00
2020	Harm reduction	1.20	21.89	132.09	962.98
2020	Specialized provider	0.31	19.41	161.65	1263.65
2021	Centralized hospital sector	1.20	19.96	143.95	246.58
2021	Decentralized hospital sector	1.20	21.02	140.80	208.75
2021	Primary health care	0.31	21.02	129.59	245.88
2021	Harm reduction	1.20	21.02	110.68	238.87
2021	Specialized provider	0.31	19.96	139.40	244.13
2022	Centralized hospital sector	1.20	19.55	86.45	242.88
2022	Decentralized hospital sector	1.20	20.58	88.85	240.82
2022	Primary health care	0.31	20.58	76.16	229.50
2022	Harm reduction	1.20	20.58	72.38	227.44
2022	Specialized provider	0.31	19.55	85.76	251.80

Table C

Mean total and Incremental Costs in 2022 USD ($) and QALYs for each hepatitis C screening strategy with a time horizon to end in 2040 and 3% discount rate. The baseline, "all treatments" accounts for the number of hepatitis C treatments that occurred in Georgia to the end of 2022 within the simulation model. For each of the care pathways, the treatments due to that pathway were removed from the total treatments for each year given in the whole elimination program to model their impact, this means that the modelled QALYs are lower, and liver disease costs higher, for each strategy compared to the base case; these incremental values are negated to represent the impact of including those treatments on QALYs gained and liver disease costs averted.

Pathway	Cost of Diagnosis ($) compared to baseline	Cost of treatments without DAAs ($) compared to baseline	Cost of treatments with DAAs ($) compared to baseline	Liver Disease Costs ($)	Total QALYs	Incremental Costs without DAAs ($)	Incremental costs with DAAs ($)	Incremental QALYs	Base Case ICER (with DAAs)*
Baseline (all treatments)	-	-	-	264,711,169	56,811,867	-	-	-	-
Hospital Centralized (4,233 treatments)	1,468,437	777,926	11,174,109	266,386,075	56,809,892	571,457	10,967,640	1,975	5752
Hospital Decentralized (5,632 treatments)	1,476,436	900,692	10,254,587	266,956,204	56,809,557	132,093	9,485,988	2,310	4258
Specialized provider (4,889 treatments)	179,229	906,958	12,594,873	266,713,412	56,809,611	-916,056	10,771,859	2,257	4948

HRP (469 treatments)	24,079	60,148	726,472	264,924,600	56,811,705	-129,204	537,121	163	3464
PHC (459 treatments)	60,891	75,390	1,016,796	264,915,505	56,811,680	-68,055	873,352	187	4870

*ICER is mean of probabilistic ICERs; USD, United States dollars; QALYs, quality adjusted life years; DAAs, direct acting antivirals; ICER, incremental cost-effectiveness ratio; HRP, harm reduction provider; PHC, primary health care

Table D

Fully incremental cost-effectiveness analysis of screening strategies for hepatitis C under different perspectives. ICER is mean of probabilistic ICERs; USD, United States dollars; QALYs, quality adjusted life years; DAAs, direct acting antivirals; ICER, incremental cost-effectiveness ratio; HRP, harm reduction provider; PHC, primary health care

Perspective	Screening pathway	Total cost compared to lowest cost scenario ($) (ordered lowest to highest)	Total QALYs compared to baseline	Incremental ICER
Base case (health system perspective)	Baseline	0	0	-
	HRP	537,121	163	3,295
	PHC	873,352	187	Extendedly dominated
	Hospital Decentralized	9,485,988	2310	4,168 [Compared to HRP]
	Specialized provider	10,771,859	2257	Dominated
	Hospital Centralized	10,967,640	1975	Dominated
Without DAA costs (provider and patient perspective)	Specialized provider	0	2257	-
	HRP	786,852	163	Dominated
	PHC	848,001	187	Dominated
	Baseline	916,056	0	Dominated
	Hospital Decentralized	1,048,149	2310	19,776 [Compared to specialized provider]
	Hospital Centralized	1,487,513	1975	Dominated
2022 costs (health system perspective for new treatment program)	Specialized provider	0	2257	-
	HRP	128,270	163	Dominated
	PHC	171,315	187	Dominated
	Baseline	179,606	0	Dominated
	Hospital Decentralized	1,239,707	2310	23,391 [Compared to specialized provider]
	Hospital Centralized	1,383,918	1975	Dominated

Fig A

Cost-effectiveness plane with DAA costs, for each of the screening pathways; Dotted line shows 20% of GDP per capita willingness to pay threshold, dashed line indicates 1 GDP per capita.

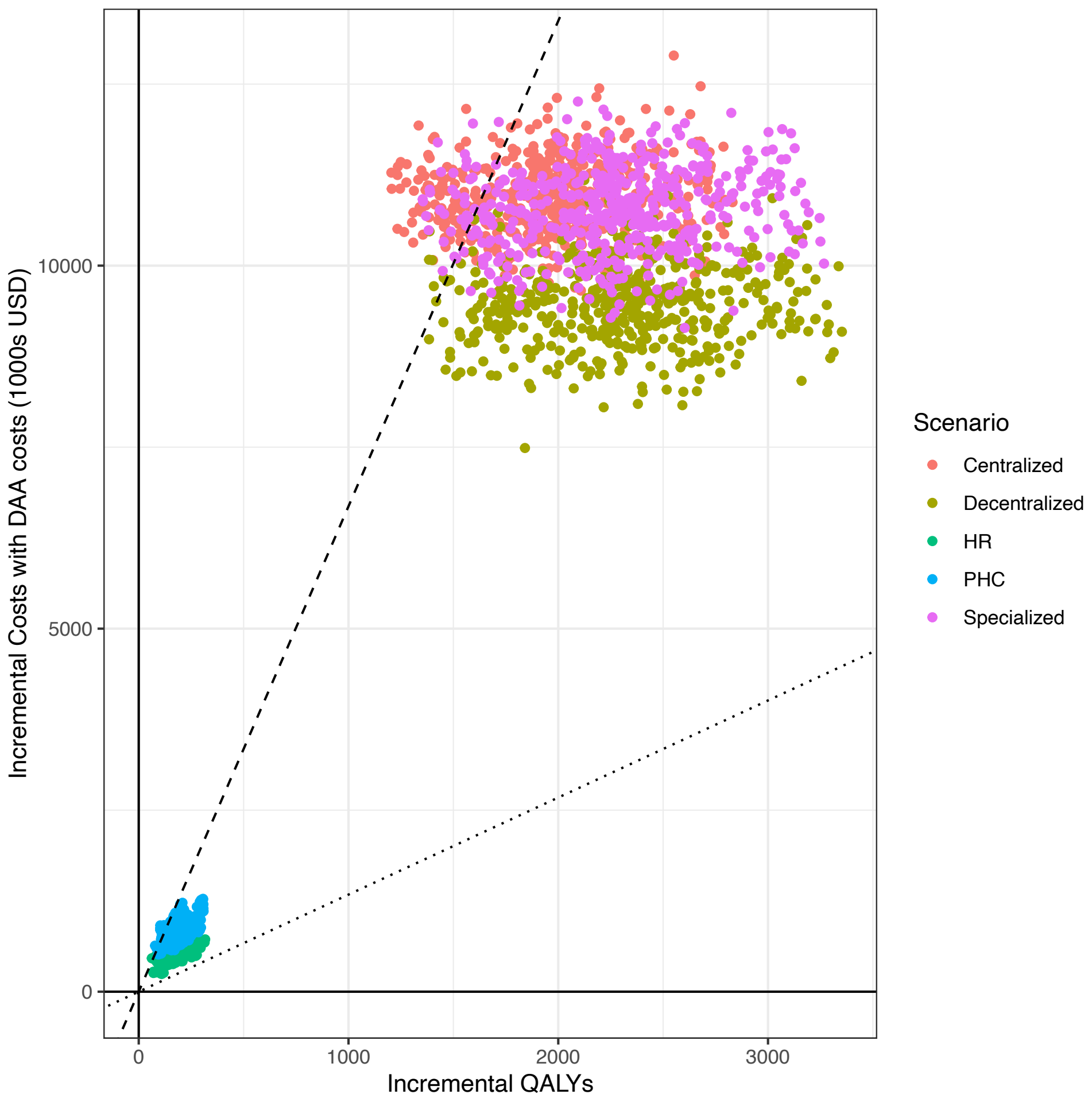

DAA, direct acting antivirals, HR, harm reduction, PHC, primary health care.

Fig B

Cost-effectiveness plane without DAA costs, for each of the screening pathways; Dotted line shows 20% of GDP per capita willingness to pay threshold, dashed line indicates 1 GDP per capita.

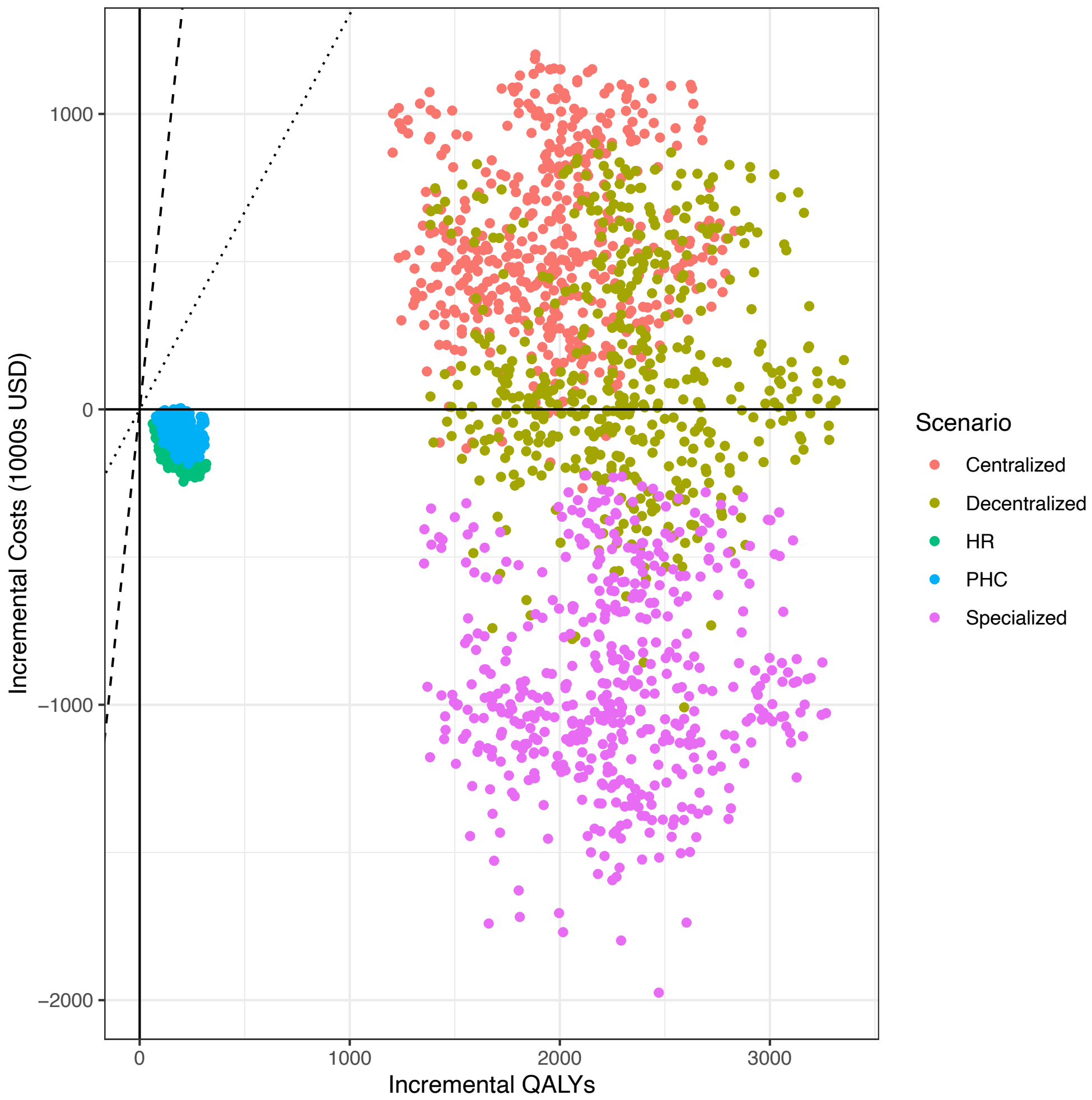

DAA, direct acting antivirals, HR, harm reduction, PHC, primary health care.
